# Brain Network Analysis and Classification Based on Convolutional Neural Network

**DOI:** 10.3389/fncom.2018.00095

**Published:** 2018-12-10

**Authors:** Lu Meng, Jing Xiang

**Affiliations:** ^1^College of Information Science and Engineering, Northeastern University, Shenyang, China; ^2^Department of Neurology, Cincinnati Children's Hospital Medical Center, Cincinnati, OH, United States

**Keywords:** convolution neural networks, brain network, word2vec, node embedding space, MEG

## Abstract

**Background:** Convolution neural networks (CNN) is increasingly used in computer science and finds more and more applications in different fields. However, analyzing brain network with CNN is not trivial, due to the non-Euclidean characteristics of brain network built by graph theory.

**Method:** To address this problem, we used a famous algorithm “word2vec” from the field of natural language processing (NLP), to represent the vertexes of graph in the node embedding space, and transform the brain network into images, which can bridge the gap between brain network and CNN. Using this model, we analyze and classify the brain network from Magnetoencephalography (MEG) data into two categories: normal controls and patients with migraine.

**Results:** In the experiments, we applied our method on the clinical MEG dataset, and got the mean classification accuracy rate 81.25%.

**Conclusions:** These results indicate that our method can feasibly analyze and classify the brain network, and all the abundant resources of CNN can be used on the analysis of brain network.

## Introduction

Brain network and brain functional/structural connectivity play an important role in neuroanatomy, neurodevelopment, electrophysiology, functional brain imaging, and neural basis of cognition (Hosseini et al., 2012). Recently, more and more graph theoretical analyses have been used to quantitatively measure the brain network of neuroimaging data. Niso et al. (2015) recorded magnetoencephalographic (MEG) data from 45 subjects (15 healthy controls and 30 migraine patients) during interracial testing state with closed eyes, and calculated 15 graph-theoretic measures to compare brain network characterizations between healthy controls and epileptic patients. Their results showed that differences in spectral power between the control and the epileptic groups have a distinctive pattern, which indicate that functional epileptic brain networks are different to those of healthy subjects during interictal stage at rest. Bassett and Bullmore (2006, 2017) analyzed the brain network using graph theoretical measures, which were clustering coefficient and path length, and concluded that the brain network had a small-world topology characterized by a high clustering coefficient between neighboring nodes and a short path length between any pair of nodes. Sherman et al. (2014) studied development of the default mode network across early adolescence based on graph theory, and found that brain network measures, such as integration, segregation, and connectivity, increased as the participants' age grow.

According to graph theory, brain networks can be composed of nodes and edges. The nodes represent neurons or brain regions, and the edges represent the physical or functional connections between nodes. Therefore, the brain network analysis using graph theory can be composed of four steps (Bullmore and Sporns, 2009): (1) define the network nodes, (2) define the measure of connections between nodes, (3) generate a adjacent matrix or undirected graph by calculating the pairwise associations between nodes, (4) calculate the graph theoretical parameters which can locally or globally characterize the brain network. Although, graph theory is widely used to analyze the brain network, there are still some shortcomings in the framework. Until now, many mathematical definitions of brain network measures have been presented, such as degree, shortest path length (Watts and Strogatz, 1998), number of triangles, global/local efficiency (Latora and Marchiori, 2001), clustering coefficient (Watts and Strogatz, 1998), transitivity (Newman, 2003), modularity (Newman, 2004), closeness centrality (Freeman, 1978), betweenness centrality (Freeman, 1978), within-module degree z-score (Guimerà and Amaral, 2005), participation coefficient (Guimerà and Amaral, 2005), anatomical and functional motifs (Milo et al., 2002; Sporns and Kotter, 2004), motif z-score(Milo et al., 2002), motif fingerprint (Sporns and Kotter, 2004), degree distribution (Barabasi and Albert, 1999), average neighbor degree (Pastor-Satorras et al., 2001), assortativity coefficient (Newman, 2002), measure of small-worldness (Humphries and Gurney, 2008). All these measures have different specific advantage and suitable for different fields, respectively, for example, shortest path length and global efficiency are suitable for measuring integration of brain network, clustering coefficient, local efficiency, and transitivity are suitable for measuring segregation of brain network, centrality and within-module degree *z*-score are suitable for measuring the centrality of brain network, motif *z*-score and motif fingerprint are suitable to measuring the brain network motifs, degree distribution, average neighbor degree, and assortativity coefficient are suitable for measuring the resilience of brain network. Therefore, different measures have different emphasis and performance on analyzing the brain network, which even result into totally conflicting results. Two studies (Leistedt et al., 2009; Zhang et al., 2011) found that major depressive disorder (MDD) patients had lower shortest path length compared with normal controls, and no significant differences in clustering coefficient. However, another study (Lord et al., 2012) found that MDD patients had a significant change of the community structures compared with healthy controls, but there was no significant differences in shortest path length and clustering coefficient.

In the last few years, convolutional neural network (CNN) has performed very well in many fields, such as image processing, artificial intelligence, human speech recognition, computer-aided diagnosis, natural language processing (NLP), and so on. The development of CNN can be tracked back in 1968, which is interestingly motivated by neuroscience findings. In 1968, Hubel and Wiesel (1968) found that cells in animal visual cortex are responsible for detecting light in receptive fields. Inspired by their findings, Kunihiko Fukushima proposed the neocognitron in 1980 (Fukushima and Miyake, 1982). Next, in 1990, LeCun et al. (1989) improved neocognitron and proposed LeNet-5 (LeCun et al., 1998), which can be recognized as the predecessor of CNN. LeNet-5 was composed of many artificial neural network layers and can be trained with backpropagation method. However, due to the poor performance of the computers at the time, the training of CNN is desperately time consuming, which means that CNN cannot resolve complicated problems at that time. As the rapid and huge development of computer hardware and software framework, as well as the Big Data technology, CNN comes back into researchers' vision again. In 2012, Krizhevsky et al. improved traditional CNN and proposed AlexNet (Krizhevsky et al., 2012), which is similar to LeNet-5 but with a deeper structure. After that, ZFnet (Zeiler and Fergus, 2014), VGGNet (Simonyan and Zisserman, 2015), GoogleNet (Szegedy et al., 2015), ResNet (He et al., 2016), etc., were proposed, all these CNN structures became deeper and deeper, and can resolve many complicated problems in image, video, and speech processing tasks. However, image, video, and speech data are represented by 1D or 2D Euclidean space discretized by rectangles, which means that CNN are suitable for these kinds of regular, grid-like, low-dimensional data. Besides Euclidean space data (image, video, speech), there are also irregular or non-Euclidean domains that can be structured with graphs, such as user data on social networks, gene data on biological regulatory networks, log data on telecommunication networks, text documents on word embeddings (Defferrard et al., 2016), as well as brain networks which is our concern in this paper.

Although, CNN has got outstanding performance in Euclidean space data, generalization of CNN to irregular or non-Euclidean data ( represented by graph) is not trivial, because the operators in CNN (convolution, pooling, Relu, dropout, etc. ) are only defined for regular grids. Analyzing the graph based on CNN is a new topic, Defferrard et al. (2016) and Kipf and Welling (2017) invoke the convolution theorem from signal processing theory and transform the graph to Fourier domain by SVD decomposition of the graph Laplacian matrix, whose eigenvalues are recognized as “frequencies” (Tixier et al., 2017). By contrast, Niepert et al. (2016) don't operate the CNN graph in the Fourier domain, they imitate the image-based convolution networks and present a general approach to extracting locally connected regions from graphs. Kawahara et al. (2017) propose novel convolution filters that leverage the topological locality of structural brain networks, in contrast to the spatially local convolutions done in the traditional image-based CNN. And they use this framework to predict clinical neurodevelopmental outcomes from brain networks.

In the present study, we aim to classified the brain network into normal group and migraine group using the MEG data from normal controls and patients with migraine. We construct the brain network using graph theory, then analyze the brain network based on CNN, instead of carefully and elaborately choosing one or several graph measures to quantitatively delineate the brain network and result in a significant difference or mathematical relationship, which may be conflicting if another measures are picked. The main contributions of the present study are: (1) build a bridge between brain network and CNN, so that the abundant CNN toolkits and methods can be used to analyze brain network; (2) the first study that classify the brain network into two categories, which are normal and abnormal (migraine); (3) represent the graph as an image and classify the graph by extracting features by building a CNN structure from the images.

## Methods

**Figure 1:**
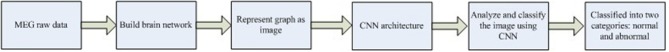
The schematic of our method.

The main steps of our method are: (1) construct a brain network using graph theory; (2) represent the graph as an image; (3) build a CNN structure; (4) analyze and classify the transformed images based on CNN. And the schematic of our method is show in Figure 1.

### Building Brain Network

Before building brain network, there are some preprocessing on the raw MEG data. Noticeable noise or artifacts were excluded using FieldTrip (an open source MATLAB toolbox, http://www.fieldtriptoolbox.org/start), and the preprocessing steps are: (1) Define segments of interest; (2) Read the MEG data (with padding) from disk; (3) Filter the data; (4) Z-transforme the filtered data and averaging it over channels; (5) Threshold the accumulated z-score.

Mathematically, brain network is represented by ordered pairs of set *G*(*N*, L) in which *N* is a set of nodes and *L* is a set of links. Graphically, the nodes are plotted as points and the links as lines joining them. When two nodes are connected by a link, they are considered neighbors (or adjacent) (Wang and Meng, 2016). In the present study, we used 275 MEG sensors as the graph nodes, and used phase lag index (PLI) (Luis et al., 2016) as the functional connections between nodes, which are graph edges.

(1) $$PLI=|\;<sign[(\Delta \varphi (t_{k}))]>$$

**Figure 2:**
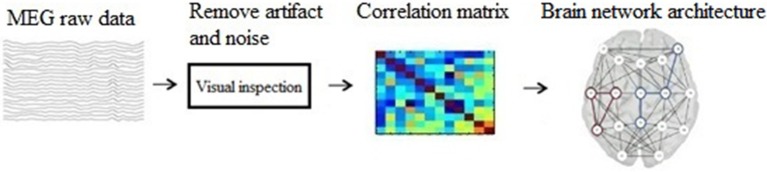
The schematic of building brain network based on graph theory.

Δ*j* represents the phase difference between two time series, *k* represents the time-point, *sign* represents signum function, < > represents the mean value and | | represents the absolute value. The schematic of building brain network is shown is Figure 2.

### Represent a Graph as an Image

This is the core contents of this study. Suppose that we have a graph *G*(*V, E*) after building the brain network, *V* represents the nodes of the graph and the element *v*_*i*_ represents the *i*th node, *E* represents the edges of the graph and the element *e*_*i, j*_ represents the weight between node *v*_*i*_ and *v*_*j*_. So the adjacency matrix *A* can be obtained, which is a square and symmetric matrix with the dimensionality of |*V*| × |*V*|, and the element *a*_*i, j*_ equals to *e*_*i, j*_. However, graph adjacency matrix does not have spatial dependence property. Therefore, we cannot directly input the graph adjacency matrix to the 2D CNN. To resolve this problem, we represent a graph as an image based on graph node embedding.

#### Graph Node Embedding

Given a graph G(V, E), a graph embedding is defined as a mapping *f* :

(2) $$f:\;v_{i}\to y_{i}\in R^{d}\;\forall i\in \left[n\right]\;and\;d\ll \;|V|$$

Therefore, the graph embedding space maps each node in the graph to a low dimensional vector, and the proximity between two nodes can be represented as the Euclidean distance between two vectors in the graph embedding space. In the present study, we calculate the mapping function *f* from graph to node embedding space, which was inspired by a NLP method, that is “Word2Vec” (Mikolov et al., 2013). Using Word2Vec method, all the words in the corpus can be represented as a low dimensional vector, instead of a high dimensional one-hot vector, and the reduction of vector dimensionality can extremely enhance the performance of many NLP tasks, such as word storage, semantic analysis, language translation, and so on. And in our study, we denoted random walk as a stochastic process which was rooted at a node *k*_0_ in the brain network and randomly chose another nodes *k*_1_,*k*_2_,…*k*_*i*_ in the neighbor of node *k*_0_. Random walk can characterize the neighboring structure of the rooted node in the brain network. Therefore, we can use a serial of random walks to illustrate the information of brain network structure, that is to say, the relationship between brain network nodes and edges. Motivated by the Word2Vec in NLP, we assume that the random walks in a brain network can be thought as sentences and phrases in a language, and all the nodes in the random walks can be though as words in a language, so we can learn not only a probability distribution of node co-occurrences, but also a representation of nodes in the format of vectors. Similarly, we name our method “Node2Vec.” The algorithm pseudo-code of Node2Vec is shown in Algorithm 1.

**Algorithm 1:** Node2Vec (*G, I, w, d, l*)

Input: (1) Graph *G*(*V, E*)
(2) Walks' iteration times *I*
(3) Window size *w*
(4) Embedding size *d*
(5) Walk length *l*
(6) Neural network set *U* = (*N*_1_*,N*_2_*,…,N*_|*V*|_*)* , each node *v*_*i*_ has one corresponding neural network *N*_*i*_, and all the networks have the same structure, that is one input layer, one hidden layer and one output layer
Output: matrix of graph nodes representations Φϵ*R*^|*V*| × *d*^
1: Initialize all the elements in *U*
2: for *i* = 0 to *I* do
3: for each *v*_*i*_ ε *V* do
4: *P* = Random Walk (*G, s, l*)
5: SkipGram (*U, P, w*)
6: end for
7: end for

Each node *v*_*i*_ in the graph has one corresponding neural network *N*_*i*_. The input layer of *N*_*i*_ is a 1 × *|V|* one-hot vector, there is only one “1” in the one-hot vector and the index of “1” indicates the specific node *v*_*i*_. The output layer of *N*_*i*_ is a 1 × *|V|* vector, and each item of vector indicates the possibility that the corresponding node is in the neighbor of node *N*_*i*_. The hidden layer of *N*_*i*_ is a 1 × *d* vector ( *d* < < |*V*| ), this is the mapping function *f* we're looking for, it means that the corresponding node in the input layer can be represented by the vector in the hidden layer, which is represented in formula (1).

Line 4 and 5 are the core steps of Node2Vec algorithm. Given a graph *G*(*V, E*), we denote random walk of length *l* rooted from node *s* as a stochastic process with random variables *X*_1_, *X*_2_,…, *X*_*l*_, such that *X*_1_ = *s* and *X*_*i*+1_ is a vertex chosen randomly from the neighbors of *X*_*i*_. We used random walk to extract local structure information from the network. The algorithm pseudo-code of random walk is shown in Algorithm 2. SkipGram algorithm maximizes the co-occurrence probability among the nodes in a window (Mikolov et al., 2013), and the algorithm pseudo-code is shown in Algorithm 3.

**Algorithm 2:** Random Walk (*G, s, l*)

Input: (1) Graph *G*(*V, E*)
(2) Starting node *s*
(3) Walk length *l*
Output: random walk path *P*_*s, l*_ = ( *X*_1_, *X*_2_,…, *X*_*l*_ )
1: Initialization: Let *i* = 1, walk length = 1, and *X*_*i*_be the starting node s
2: while ( walk length hasn't reached *l* )
3: Let node *X*_*i*+1_ be a random neighbor of node *X*_*i*_
4: Add node *X*_*i*+1_ in the random walk path *P*_*s, l*_
5: Let walk length added by 1
6: end while

**Algorithm 3:** .SkipGram (*U, P, w*)

Input: (1) Neural network set *U* = (*N*_1_*,N*_2_*,…,N*_|*V*|_*)*
(2) Random walk path *P*
(3) Window size *w* Output: update the neural network set *U*
1: for each *v*_*j*_ϵ*P* do
2: for each *u*_*k*_ ϵ *P*[*j*-*w*: *j*+*w*] do
3: Represent *v*_*j*_ by a one-hot vector *h*_*j*_
4: Let *h*_*j*_ be the input layer of *N*_*j*_
5: Update output layer using *u*_*k*_
6: Train neural network *N*_*j*_, and update the hidden layer of *N*_*j*_
7: end for
8: end for

#### Represent Graph as Image

In the graph node embedding space, we obtain the matrix of graph nodes representations Φϵ*R*^|*V*| × *d*^, so we can represent each node *v*_*i*_ in the graph as a *d*-dimension vector. And in the viewpoint of machine learning, we can also conclude that each node *v*_*i*_ in the graph has *d* features. Next, we need to align all these features to determine which feature is the most important one, which dimensional is the second important one, and so on. Therefore, in this paper, principle component analysis (PCA) method is used to transform the *d*-dimension vector of node *v*_*i*_ into *d*_*PCA*_-dimension vector *L*_*PCA*_, which is a sequential list.

(3) $$L_{PCA}=\left\{f_{1},f_{2},\ldots ,f_{d_{PCA}}\right\}\;1\leq i\leq \left|V\right|\;,\;d_{PCA}<d$$

*|V|* represents the number of nodes in the graph. Then, we can build a matrix *M*_1_ using the first two features of all nodes,

(4) $$M_{1}=\left|\begin{array}{c} f_{1}^{v_{1}},f_{1}^{v_{2}},f_{1}^{v_{3}},\ldots ,f_{1}^{v_{\left|V\right|}}\\ f_{2}^{v_{1}},f_{2}^{v_{2}},f_{2}^{v_{3}},\ldots ,f_{2}^{v_{\left|V\right|}} \end{array}\right|$$

$f_{1}^{v_{i}}\left(1\leq i\leq |V|\right)$ represents the first feature of node *v*_*i*_, $f_{2}^{v_{i}}\left(1\leq i\leq |V|\right)$ represents the second feature of node *v*_*i*_. Then we normalize $f_{1}^{v_{i}}$ and $f_{2}^{v_{i}}$ to a fixed number *r* of equally-size bins. Then we can build the image *I*_1_, the resolution of the image *I*_1_ is *r* × *r*, and the value of image pixels are defined as the count of the number of nodes falling into that bin. The algorithm pseudo-code is shown in Algorithm 4.

**Algorithm 4:** Graph2Img (*U, P, w*)

Input: (1) Graph *G*(*V, E*)
(2) Matrix of graph nodes representations Φϵ*R*^|*V*| × *d*^
Output: Image *I*_1_ representing graph
1: Initialization: Let all pixels of *I*_1_ to be zero
2: Re-organize *?* by PCA method
3: Build a matrix *M*_1_ using the first two features of all nodes
4: for *i* = 1 to |V| do
5: x = $f_{1}^{v_{i}}$
6: y = $f_{2}^{v_{i}}$
7: Let *I*_1_ (*x, y*) added by 1
8: end for

**Figure 3:**
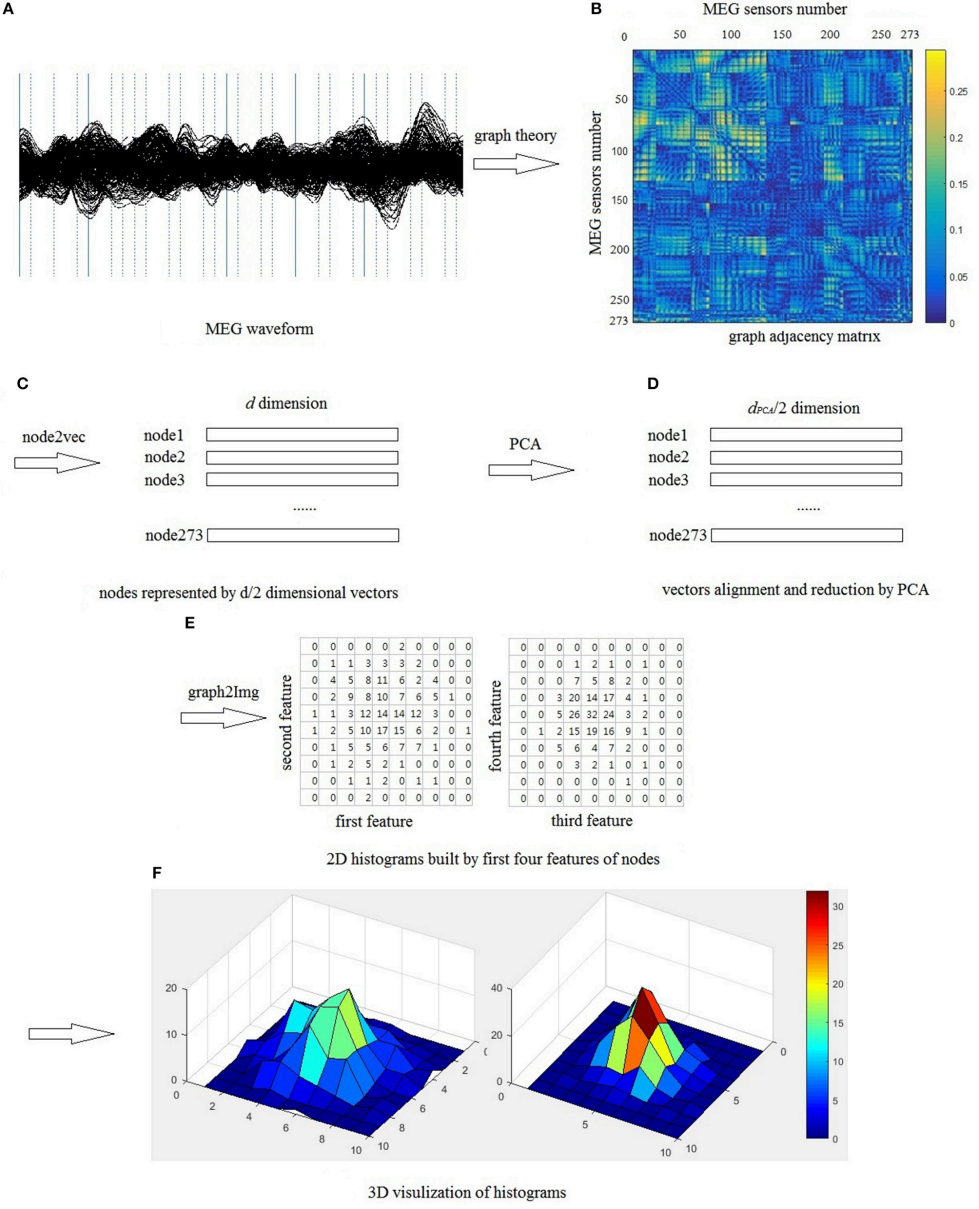
Schematic of representing graph as images, **(A)** segments of MEG waveform from a healthy control, **(B)** use 273 sensors as nodes, PLI as edges to build brain network, **(C)** each in the graph is represented by a *d*/2 dimensional vector, **(D)** use PCA method to align and reduce the vectors, **(E)** build 2D histogram, in this study, each feature is divided into ten bins, therefore, each value in the 10 × 10 matrix is the number of nodes falling into the corresponding bin, and the sum of all pixel values is 273, which is the number of sensors, **(F)** 3D visualization of histogram.

Similarly, we also can build image *I*_2_ using the third and fourth features of all nodes, build image *I*_3_ using the fifth and sixth features of all nodes, and so on. Totally, we can build *d*_*PCA*_/2 images from the graph node embedding space. However, we don't have to use all the *d*_*PCA*_ /2 images, because PCA method is used to reduce and align the features into a sequential list. In this study, we only use the first four features to build two images *I*_1_ and *I*_2_, which is enough to analyze and classify the brain network, shown in Figure 3.

### CNN Architecture

**Figure 4:**
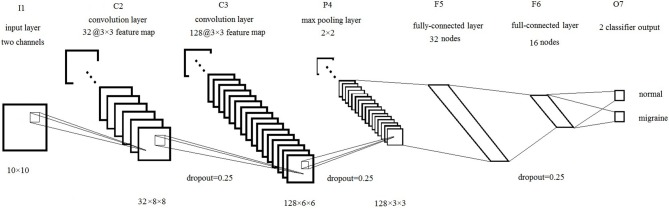
Schematic representation of CNN structure.

The brain network is represented by two images *I*_1_ and *I*_2_. We can also recognize *I*_1_ and *I*_2_ as two channels of one image, just like (R,G,B) channels in the color images. Then we can use these images as an input to CNN. In this study, our CNN structure is based on LeNet-5 (LeCun et al., 1998), there are seven layers totally, including input layer (I1), convolution layer 1 (C2), convolution layer 2 (C3), pooling layer (P4), full connection layer 1 (F5), full connection layer 2 (F6), and output layer (F7), shown as Figure 4.

Firstly, the input layer is a 10 × 10 image. Then, the first convolution layer is 32 kernels of 3 × 3 feature map, each kernel computes a convolution of the input image with a ReLU activation function. The second convolution layer is 128 kernels of 3 × 3 feature map with a ReLU activation function, followed by a 2 × 2 max pooling layer. Next, there two fully-connected layers with 32 nodes and 16 nodes. Finally, the output layer with two output nodes is used to classify the input images into two categories based on the softmax function.

## Experiment and Result

In this section, we applied our method on the clinical MEG dataset, which is consists of 40 subjects, 20 healthy controls (subject ID: 1 to 20) and 20 patients with migraine (subject ID: 21–40). All these MEG data were obtained from Cincinnati Children's Hospital Medical Center (CCHMC) and Nanjing Brain Hospital, and the target is to classify MEG data into two classes: the abnormal subjects and the healthy controls.

Our method use some open-source toolkits, MEG data is pre-processed by using FieldTrip toolkit (Oostenveld et al., 2011), brain network is built by using FieldTrip toolkit, graph node embedding is calculated by using node2vec toolkit (Grover and Leskovec, 2016), PCA is performed by using Matlab PCA toolkit, histogram of vectors is calculated by using histograms python toolkit (Tixier et al., 2017), our CNN is implemented using the Keras model with tensorflow (Abadi et al., 2016) backend. All these algorithms are run on Intel Core i7-6700 3.4 GHz CPU and 8 GB of RAM, under Windows 7 × 64 operating system and Python 3.5.

In this study, brain networks of MEG data are represented as images with two channels and resolution 10 × 10. We perform 4-fold cross-validation; the 40 MEG datasets are randomly split into four equal size subsamples. In each run, three subsamples are selected as the train grouping, and the remaining single subsample is retained as the test group. Input all these images of train group into our CNN architecture, and the categorical cross-entropy loss is optimized with Adam. To avoid over-fitting, dropout = 0.25 is used after convolution layers C2, C3 and fully-connected layer F5, and early stopping is also used after every epoch, so the number of epochs of each run is different. The training parameters are as following: dropout rate = 0.25, regularization weight = 5 × 10^−4^, learning rate = 0.001, momentum = 0.1, training epoch = 1,000, iteration = 10. After training from CNN, the filter kernels of convolution layers (C2, C3) and the weights of full-connected layers (F5, F6) can be determined, then the training results can be used to validate the test group.

**Figure 5:**
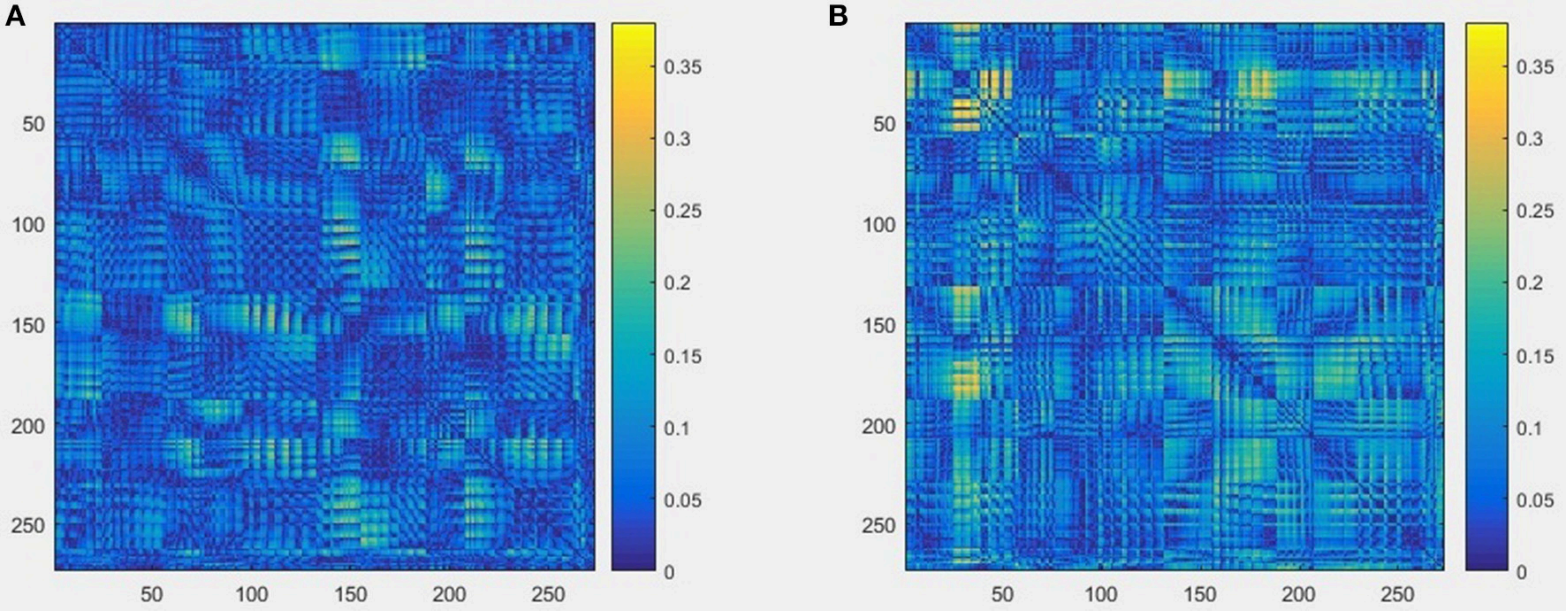
An example of brain network result built from raw MEG data, and the size is 273 × 273, **(A)** one healthy control (subject ID = 10), **(B)** one patient with migraine (subject ID = 30).

**Figure 6:**
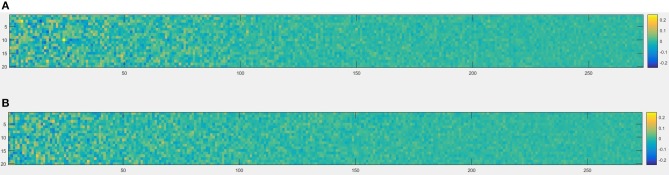
An example of node2vec result built from brain network, each node of the brain network was represented as a 20-dimensional vector in the node embedding space, so the size was 273 × 20, **(A)** one healthy control (subject ID = 10), **(B)** one patient with migraine (subject ID = 30).

**Figure 7:**
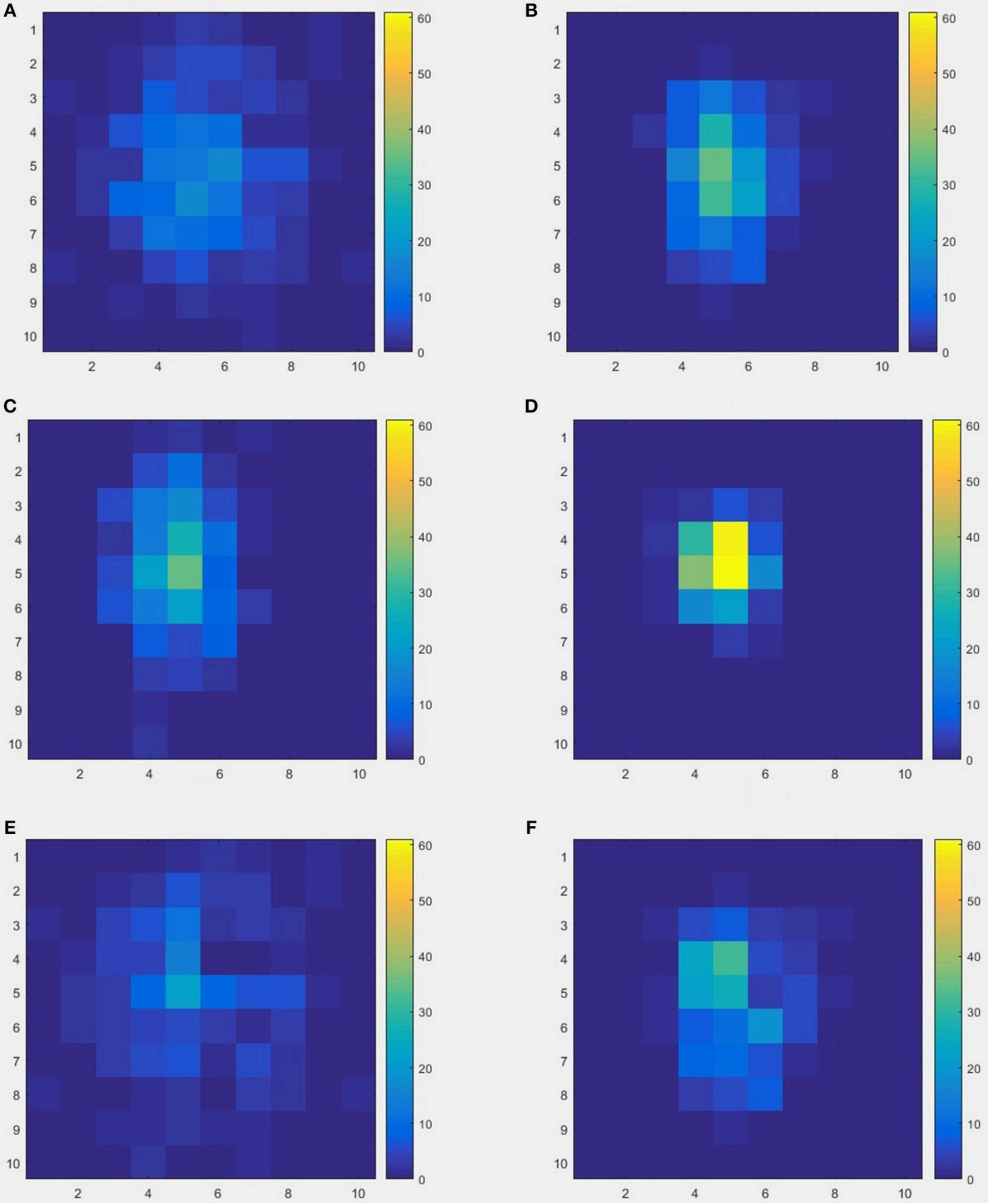
An example of two-channel images results by from 20-dimensional node vectors, each subject was represented as an image with two channels, so the image size is 10 × 10 × 2, **(A,B)** represent the two channels from one healthy control (subject ID = 10), **(C,D)** represent the two channels from one patient with migraine (subject ID = 30), **(E,F)** represent the differences between healthy control and patient.

The schematics of the whole procedure of one healthy control (subject ID = 10) and one patient with migraine (subject ID = 30) were shown in Figures 5, 7. And we can see that it's not clear to distinguish the differences between healthy controls and patients with migraine in the brain network (Figure 5) or in the node embedding space (Figure 6), however, in the representation of two-channel images (Figure 7), the differences can be easily found and classified by CNN.

**Figure 8:**
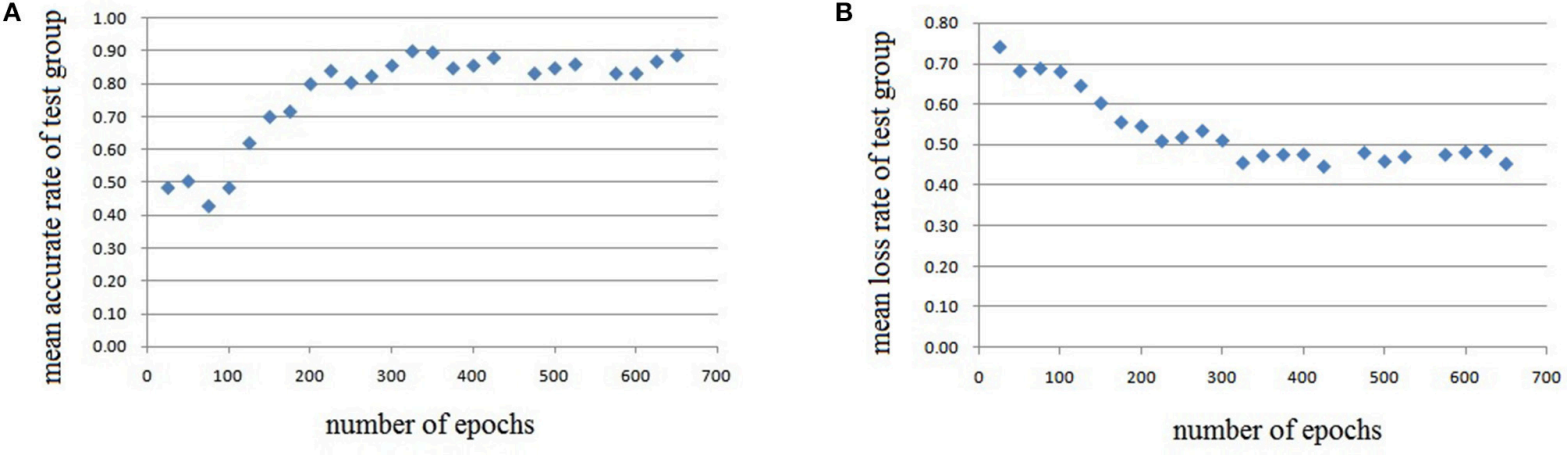
Testing results of the first run, each dot in the image represents the mean value of every 25 epochs, **(A)** mean accurate rate of test group, **(B)** mean loss rate of the group.

**Figure 9:**
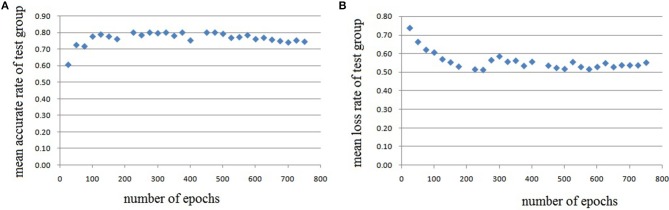
Testing results of the second run, each dot in the image represents the mean value of every 25 epochs, **(A)** mean accurate rate of test group, **(B)** mean loss rate of the group.

**Figure 10:**
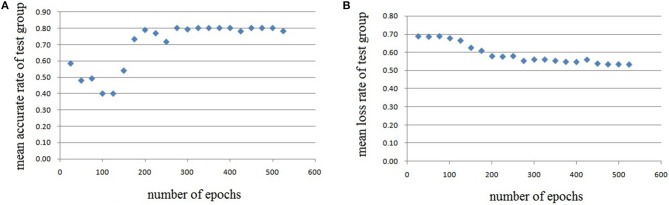
Testing results of the third run, each dot in the image represents the mean value of every 25 epochs, **(A)** mean accurate rate of test group, **(B)** mean loss rate of the group.

**Figure 11:**
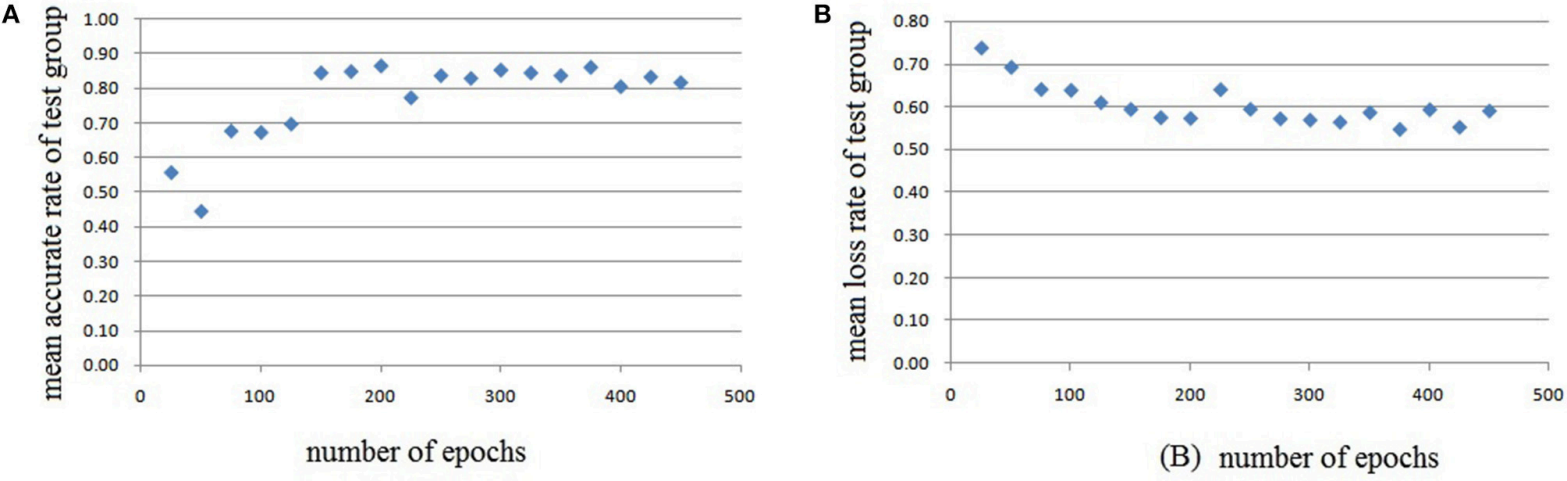
Testing results of the fourth run, each dot in the image represents the mean value of every 25 epochs, **(A)** mean accurate rate of test group, **(B)** mean loss rate of the group.

**Figure 12:**
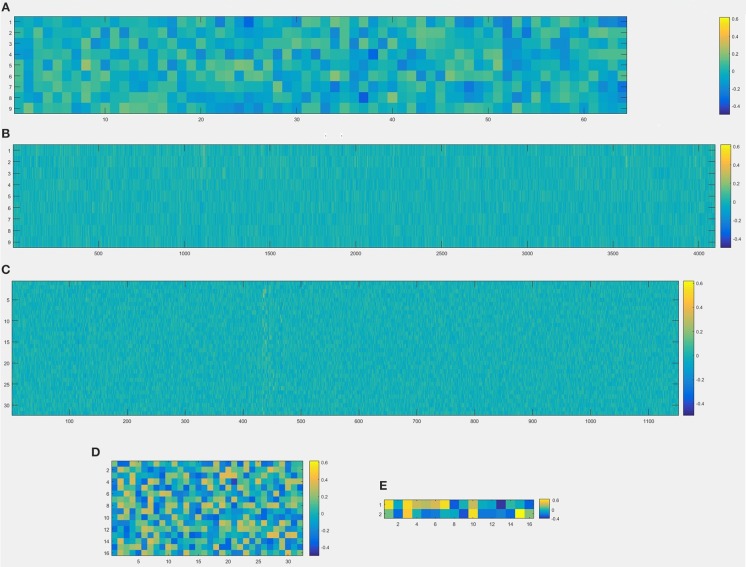
Visualization of weights in all layers from convolutional neural network, **(A)** the first convolve layer with size (3, 3, 2, 32), which means that there are 64 kernels whose size are 3 × 3, and this layer is visualized by a 9 × 64 image; **(B)** the second convolve layer with size (3, 3, 32, 128), which means that there are 4,096 kernels whose size are 3 × 3, and this layer is visualized by a 9 × 4096 image; **(C)** the first fully-connected layer with size (1152, 32), which means that 1,152 units from the output of the last layer and 32 units from the input of first fully-connected layer, and this layer is visualized by a 1,152 × 32 image; **(D)** the second fully-connected layer with size (32, 16), which means that 32 units from the output of the last layer and 16 units from the second fully-connected layer, and this layer is visualized by a 32 × 16 image; **(E)** the third fully-connected layer with size (16, 2), which means that 16 units from the last layer and 2 classifications as the output result (normal or migraine), and this layer is visualized by a 16 × 2 image.

In this experiment, we repeat the training and testing procedures ten iterations to ensure the stability of the method. Limited by the article length, we can't show all the results from 10 iterations. Therefore, from the iteration #1 of 4-fold cross-validation, we illustrate the validation accuracy rate of every 25 epochs and validation loss rate of every 25 epochs, which are shown in Figures 8–11. The accuracy rate and loss rate were steady after 300 epochs. The classification accuracy rate of four cross-validation are shown in Table 1. Therefore, we may conclude that our method can analyze and classify the brain network into two categories: normal and migraine. After training of 10 iterations, we can obtain the model and weights of each layer. Visualization of weights in all layers from our CNN is shown in Figure 12. Figure 12A shows the weights of the first convolve layer with size (3, 3, 2, 32); Figure 12B shows the weights of the second convolve layer with size (3, 3, 32, 128); Figure 12C shows the weights of the first fully-connected layer with size (1152, 32); Figure 12D shows the weights of the second fully-connected layer with size (32, 16); Figure 12E shows the weights of the third fully-connected layer with size (16, 2). By the visualization, we can see that our convolution kernel size is only 3 × 3, we didn't choose the bigger one, because the resolution of our target is 10 × 10, and large kernel may hinder feature extraction by the feature maps. However, as a remedy for the small kernel size, we used large amount of feature maps and units in the convolve layers and fully-connected layers, and the total number of parameters in our CNN is 74,784, which can make sure that the differences between healthy controls and patients with migraine can be extracted by our CNN.

**Table 1:** Results of accuracy rate from four cross-validation in iteration #1.

	**First cross-validation (%)**	**Second cross-validation (%)**	**Third cross-validation (%)**	**Forth cross-validation (%)**	**Mean**
Accuracy rate	88.9	74.6	79.0	82.5	81.25%

Besides, we also compared our classification results with the other two base line methods, Linear SVM and graph convolution neural network (GCNN) from Defferrard et al. (2016), shown in Table 2. Linear SVM is a classical supervised learning method for classification and regression analysis, but for the classification of MEG raw data, the mean accuracy rate is only 58.37%, we guess the poor performance is due to the huge amount of MEG channels, and multiple dimensionalities of MEG data. GCNN and our method both outperform Linear SVM, which indicates that the integration of graph theory and CNN can greatly enhance the performance of classification accuracy, however, GCNN has not made special optimization for MEG data, so it lags behind our method in classification accuracy.

**Table 2:** The comparison of our method and two baseline methods.

**Method**	**Mean accuracy rate (%)**
Linear SVM	58.37
GCNN	75.60
Our method	81.25

## Conclusion and Future Work

In this paper, we bridge the gap between brain network and convolution neural network, and classify the brain network from MEG data into two categories: normal and migraine. We train the CNN architecture on the training group, and validate the result on the testing group, which indicates that our method is feasible and can distinguish normal and migraine brain network.

Next, we will mainly focus on two aspects: (1) collect more MEG data, and improve the CNN architecture; (2) diversify the abnormal MEG brain network, and use our method on the epileptic brain network, autism brain network, and so on; (3) analyze the brain network at the source level.

## Author Contributions

LM corresponding author, main contributor. JX data provider and professional advisor.

## Conflict of Interest Statement

The authors declare that the research was conducted in the absence of any commercial or financial relationships that could be construed as a potential conflict of interest.
